# Next-generation sequencing in thyroid cancer

**DOI:** 10.1186/s12967-016-1074-7

**Published:** 2016-11-21

**Authors:** Yoon Jin Cha, Ja Seung Koo

**Affiliations:** Department of Pathology, Yonsei University College of Medicine, 50-1 Yonsei-ro, Seodaemun-gu, Seoul, 03722 South Korea

**Keywords:** Next-generation sequencing, Cancer, Cytology, Thyroid

## Abstract

Next-generation sequencing (NGS) in thyroid cancer allows for simultaneous high-throughput sequencing analysis of variable genetic alterations and provides a comprehensive understanding of tumor biology. In thyroid cancer, NGS offers diagnostic improvements for fine needle aspiration (FNA) cytology of thyroid with indeterminate features. It also contributes to patient management, providing risk stratification of patients based on the risk of malignancy. Furthermore, NGS has been adopted in cancer research. It is used in molecular tumor classification, and molecular prediction of recurrence and metastasis in papillary thyroid carcinoma. This review covers previous NGS analyses in variable types of thyroid cancer, where samples including FNA cytology, fresh frozen tissue, and formalin-fixed, paraffin-embedded tissues were used. This review also focuses on the clinical and research implications of using NGS to study and treat thyroid cancer.

## Background

An understanding of the molecular mechanisms of tumor formation is mandatory for accurate diagnoses and personalized treatments. Previously, single gene assays were commonly used for finding molecular alterations in tumors. Presently, NGS technology provides the simultaneous analysis of hundreds of genes of interest, using targeted sequencing panels [1]. Thus, NGS-based molecular tests for oncology research and clinical practice appear to be rapidly evolving.

Thyroid cancer is the most common malignancy of the endocrine organs; its prevalence is increasing, more than tripling during the last three decades [2]. Papillary thyroid carcinoma (PTC) is the most common type of thyroid cancer, followed by follicular carcinoma, medullary carcinoma, poorly differentiated carcinoma, and anaplastic carcinoma. NGS assays can allow improvements in diagnostic accuracy and precise personalized treatments. Thyroid cancers harbor characteristic genetic alterations, including point mutations for proto-oncogenes (*BRAF, NRAS, HRAS, KRAS*) and chromosomal rearrangements (*RET/PTC1, RET/PTC3, PAX8/PPARG*), which vary with histologic subtype [3]. This review outlines the results of NGS assays in thyroid cancer, and highlights their clinical implications.

## NGS application in the diagnosis of indeterminate cytology

A majority of previous studies using NGS in thyroid cancer analyzed variable specimen sample types and histologic subtypes (Table 1). In clinical practice, NGS assays have been used in the diagnosis of indeterminate cytology of thyroid nodules. FNA is an efficient method for evaluating thyroid nodules that has high sensitivity and specificity. However, FNA has some limitations, since 20–30% of FNA samples fall into categories of indeterminate cytology. These categories include atypia of undetermined significance/follicular lesion of undetermined significance (AUS/FLUS, category III); follicular or oncocytic (Hurthle cell) neoplasm/suspicious for a follicular or oncocytic (Hurthle cell) neoplasm (FN/SFN, category IV); and suspicious for malignant cells (SUSP) [4–8]. The average cancer risk for these categories is 15.9% in AUS/FLUS, 26.1% in FN/SFN, and 75.2% in SUSP [9]. NGS contributes to diagnostic decision making in patients with indeterminate cytology. Findings from previous studies, which used NGS to analyze thyroid nodules with indeterminate cytology, are summarized in Table 2.

**Table 1:** Studies of thyroid cancer using next-generation sequencing platforms

Author (year)	Diagnosis	Patient number	Sample type	Platform	Mutational panel	Analytic process tool	Important findings
Nikiforova et al. [10]	Classic PTC	27	Fresh frozen tissue FFPE tissue	Ion Torrent PGM	ThyroSeq	Torrent Suit version 3.4.2	1. 70% of classic PTCs harboring mutations: *BRAF* (59%) > *PIK3A* (11%) > *TP53* (7%) > *NRAS* (4%)
	FVPTC	30					
							2. 83% of FVPTC harboring mutations: *RAS* (73%) > *BRAF* (7%) > *TSHR* (3%)
	Classic FC	18					
							3. Mutations of FC in order of frequency Conventional type: *NRAS* > *TSHR* > *KRAS* Oncocytic type: *TP53* > *HRAS* > *KRAS* > *PTEN*
	Oncocytic FC	18					
							4. 30% of PDCs harboring mutations of *NRAS*, *PIK3CA*, *GNAS*, *BRAF* and 74% of ACs harboring mutations of *TP53*, *BRAF*, *RAS*, *PIK3CA*, *PTEN*, *CTNNB1*
							5. 11 MCs (73%) harboring mutations: 7 *RET* (47%), 3 *HRAS* (20%), 1 *KRAS* (7%)
	PDC	10					
	AC	27					
	MC	15					
Smallridge et al. [11]	PTC, BRAF-mutant	12	Fresh frozen tissue	Illumina HiSeq 2000	RNA-Seq (13085 genes)	TopHat tool package	1. 51 genes related with immune function pathway are downregulated in *BRAF* V600E-mutant PTCs
							2. *HLAG, CXCL14, TIMP1, IL1RAP* are overexpressed in BRAF V600E-mutant PTCs mutation
	PTC, BRAF-wild type	8					
Leeman-Neil et al. [12]	PTC, radiation-associated	62	Fresh frozen tissue	Illumina HiSeq 2000	RNA-Seq	ChimeraScan and defuse program	ETV6-NTRK3 rearrangement are present in 14.5% of radiation-associated PTCs and 2% of sporadic PTCs
	PTC, sporadic	151					
Simbolo et al. [32]	MC	20	FFPE tissue	Ion Torrent PGM	Ion AmpliSeq Hot Spot Cancer Panel v2 (50 genes)	Torrent Suit version 3.6	1. 85% of MCs have mutations: 13 *RET* (60%), 3 *HRAS* (15%), 1 *KRAS* (5%), 1 *STK11* (5%), and 3 undetected (15%)
							2. Three *RET* mutations are found by NGS test, which are negative by Sanger sequencing
Nikiforov et al. [34]	FN/SFN	143 (retrospective group, *N* = 91; prospective group, *N* = = 52)	FNA	Ion Torrent PGM	ThyroSeq v2	N/A	Performance of NGS test in cancer detection among nodules with FN/SFN cytology: sensitivity 90%, specificity 93%, PPV 83%, NPV 96%, accuracy 92%
Cancer genome atlas research network [13]	PTC	496	Fresh frozen tissue	Illumina HiSeq 2000	Whole genome sequencing	Picard pipeline	1. Identification of potential new tumor-initiating mutation in PTC lacking known driver mutation (*EIF1AX, PPMID, CHEK2*)
							2. *TERT* promoter mutation (1%) associated with high risk of recurrence
							3. Categorization of PTC into *BRAF*-like and *RAS*-like types based on the multi-level molecular data
Sykorova et al. [27]	PDC	3	Fresh frozen tissue	Illumina MiSeq	The TruSight Cancer Panel (94 genes)	MiSeq Reporter v.2.4	1. All PDC and AC harbor more than one genetic alteration, and *TP53* mutation is commonly present except for 2 cases
							2. Altered genes in PDCs: *CDH1, FANCD2, CHECK2, ADH1B, GPC3, TP53*, *PTEN*
							3. Altered genes in ACs: *ATM, HNF1A, MET, NF1, TP53, PTEN, MSH2, RB1, NBN, NF1, MUTYH, TSC2, HRAS, EGFR*
	AC	5					
Le Mercier et al. [35]	Indeterminate cytology	34	Cell blocks (FFPE) and smears	Ion torrent PGM	AmpliSeq Cancer hotspot panel version 2	Torrent Suit version 3.6.2	Performance of NGS test in cancer detection among nodules with indeterminate cytology: sensitivity 71%, specificity 89%, PPV 62%, NPV 92%, accuracy 85%
					Ion AmpliSeq HiFi Master Mix	Variant Caller plugin version 3.6	
Nikiforov et al. [44]	AUS/FLUS	465	FNA	Ion torrent PGM Ion proton	ThyroSeq v2.1	N/A	Performance of NGS test in cancer detection among nodules with AUS/FLUS cytology: sensitivity 90.9%, specificity 92.1%, PPV 76.9%, NPV 97.2%, accuracy 91.8%
Picarsic et al. [14]	PTC	17 (age <18 years)	FNA Fresh frozen tissue FFPE tissue	Ion Torrent PGM	ThyroSeq v2 (14 gene and 42 types gene fusions)	N/A	1. Mutation detection rate is increased from 60% with 7-gene mutation panel to 80% with NGS 2. Chromosomal rearrangement is more common than point mutation in pediatric PTC (53 vs. 33%) 3. *ETV6*-*NTRK3* fusions are present in 18% and associated with unfavorable histology such as solid, insular, or trabecular patterns
Ballester et al. [15]	PTC	25 (age range, 10–19 years)	FNA FFPE tissue	Ion torrent PGM	AmpliSeq Cancer Hotspot Panel v2 (50 genes)	Torrent Suit version 4.2	No additional mutation detected by NGS in cases lacking mutations in *BRAF, RET/PTC, TERT* promoter mutation at initial analysis
Landa et al. [28]	PDC	34	Fresh frozen tissue (*N* = 37) FFPE tissue (*N* = 80)	N/A	MSK-IMPACT cancer exome panel (341 genes)	MSK-IMPACT pipeline	1. Mutation number is greater in AC (6 ± 5) than PDC (2 ± 3), and predominantly affected genes are *TP53*, *TERT* promoter, PI3K/AKT/mTOR pathway effector, SWI/SNF subunit, and histone methyltransferase
							2. 92% of *RAS* mutations are found in PDCs met Turin criteria; 81% *BRAF* mutations are present in PDCs met MSKCC criteria
							3. *BRAF*-mutant PDCs are smaller and frequently metastasize to lymph nodes; RAS-mutant PDCs are larger and have higher frequency of distant metastasis
							4. *EIF1AX* mutations are detected in 11% of PDCs and 9% of ACs, and 93% of which are associated with RAS mutations
							5. Chromosomal rearrangement is detected in 14% of PDCs (*RET*/PTC, *PAX8/PPARG*, and *ALK/EML4*) and absent in AC
	AC	33					
Swierniak et al. [20]	FA	26	Fresh frozen tissue	Illumina HiSeq 1500	TruSeq panel (372 genes)	Varscan2 CODEX package BreakDancer FACTERA	1. Common somatic alterations: oncogenes (*MDM2*, *FLI1*), transcription factors and repressors (*MITF, FLI1, ZNF331*), epigenetic enzymes (*KMT2A, NSD1, NCOA1, NCOA2*), and protein kinases (*JAK3, CHEK2, ALK*) 2. Single nucleotide variant is the most common and large structural variants are the least 3. Identification of novel translocation, *DERL/COX6C*
	FC	20					
	PDC	2					
	Paired normal thyroid tissue	34					

*AUS*/*FLUS* atypia of undetermined significance/follicular lesion of undetermined significance, *FNA* fine needle aspiration, *N*/*A* not applicable, *PPV* positive predictive value, *NPV* negative predictive value, *FN*/*SFN* follicular or oncocytic (Hurthle cell) neoplasm/suspicious for a follicular or oncocytic (Hurthle cell) neoplasm, *FFPE* formalin-fixed, paraffin-embedded, *PTC* papillary thyroid carcinoma, *FVPTC* follicular variant papillary thyroid carcinoma, *FC* follicular carcinoma, *PDC* poorly differentiated carcinoma, *AC* anaplastic carcinoma, *MSKCC* Memorial Sloan-Kettering Cancer Center, *MC* medullary carcinoma

**Table 2:** Application of next-generation sequencing in the thyroid nodule with indeterminate cytology

Author (year)	Sample type	Patient number	Histologic diagnosis	Platform	Mutational panel	Analytic process tool	Sensitivity (%)	Specificity (%)	PPV (%)	NPV (%)	Accuracy (%)
Nikiforov et al. [34]	FNA	143	Retrospecfic cohort (*N* = 91): 27 mutation positive: 2 PTC, 18 FVPTC, 3 FC, 2 FA, 2 HN Prospecfive cohort (*N* = 52): 15 mutation positive: 1 PTC, 8 FVPTC, 3 FC, 2 FA, 1 HN	Ion Torrent PGM	ThyroSeq v2	N/A	90	93	83	96	92
Le Mercier et al. [35]	Cell blocks (FFPE) and smears	34	8 mutation positive: 1 PTC, 1 FVPTC, 2 MIFC, 1 FTUMP, 3 FA	Ion Torrent PGM	AmpliSeq Cancer hotspot panel version 2	Torrent Suit version 3.6.2	71	89	62	92	85
					Ion AmpliSeq HiFi Master Mix	Variant Caller plug-in version 3.6					
Nikiforov et al. [44]	FNA	465	96 nodules resected 31 mutation positive: 2 PTC, 18 FVPTC, 4 FA, 2 HN	Ion Torrent PGM and Ion proton	ThyroSeq v2.1	N/A	91	92	77	97	92

*PPV* positive predictive value, *NPV* negative predictive value, *FNA* fine needle aspiration, *PTC* papillary thyroid carcinoma, *FVPTC* follicular variant papillary thyroid carcinoma, *FA* follicular adenoma, *HN* hyperplastic nodule, *N*/*A* not applicable, *FFPE* formalin-fixed, paraffin-embedded, *MIFC* minimally invasive follicular carcinoma, *FTUMP* follicular tumor with uncertain malignant potential, *MNG* multinodular goite

## Genetic alteration of thyroid cancer and NGS

### Papillary carcinoma

Several studies have applied NGS to variable subgroups of PTCs. Nikiforova et al. analyzed FFPE or frozen tissue from 27 classic PTCs and 30 FVPTCs, using the ThyroSeq NGS panel targeting 12 genes with 34 amplicons on the Ion Torrent PGM sequencer [10]. The results showed that 70% of classic PTCs harbored mutated genes: *BRAF* (59%) was the most frequent, followed by *PIK3A* (11%), *TP53* (7%), and *NRAS* (4%). In contrast, 83% of FVPTCs had mutated genes: *RAS* (73%) was the most frequent, followed by *BRAF* (7%) and *TSHR* (3%) [10]. Smallridge et al. performed RNA sequencing (RNA-Seq) using the Illumina HiSeq 2000 platform on frozen tissue from 12 *BRAF* V600E-mutated PTCs and 8 *BRAF*-wild type PTCs [11]. Among the 13,085 genes interrogated, 560 were differentially expressed between *BRAF* V600E-mutated PTCs and *BRAF*-wild type PTCs [11]. Among these 560 genes, 67 were related to immune function pathways, 51 were under-expressed in *BRAF* V600E-mutated PTCs, and *HLAG, CXCL14, TIMP1,* and *IL1RAP* were over-expressed. In *BRAF*-wild type PTCs, 4 immune function genes (*IL1B, CCL19, CCL21,* and *CXCR4*) were most significantly differentially expressed, and exhibited a high degree of correlation with lymphocytic infiltration [11]. In a study by Leeman-Neill et al. fresh frozen tissue from 62 radiation-associated PTCs and 151 sporadic PTCs was analyzed using RNA-Seq on the Illumina HiSeq 2000 platform. This identified an *ETV6*-*NTRK3* rearrangement in 14.5% of radiation-associated PTCs and 2% of sporadic PTCs [12]. The authors suggested that an *ETV6*-*NTRK3* rearrangement may be a key mechanism of radiation-induced carcinogenesis [12].

The Cancer Genome Atlas (TCGA) Network described the genomic characterization of 496 PTCs, and generated data using whole genome sequencing. This was done on the NGS platform and by a multiplatform analysis of SNP arrays, DNA methylation, and reverse phase protein arrays [13]. In PTCs that lacked known driver mutations, alterations of *EIF1AX, PPMID,* and *CHEK2* were discovered as potential new tumor-initiating mutations. The TCGA project identified the *TERT* promoter mutation, which accounts for approximately 1% of PTCs, but shows association with a high risk of recurrence. Based on the multi-level molecular data, PTCs were separated into two groups of distinct downstream signaling pathways: the BRAF^V600E^-like cohort and the RAS-like cohort. Genomic, epigenomic, and proteomic differences were revealed between these two groups, and most of the RAS-like PTCs were follicular variant PTCs (FVPTCs).

Regarding pediatric thyroid carcinoma, Picarsic et al. analyzed 17 pediatric PTCs (age range 8–17 years, median 13 years) from FNA, fresh frozen tissue, and FFPE samples. Mutation analysis with a 7-gene mutation panel using real-time PCR and ThyroSeq v2 on the Ion Torrent PGM sequencer showed that: (1) The detection rate of molecular alterations was increased by up to 87% by the ThyroSeq v2 NGS assay compared to an increase of 60% by the 7-gene mutation panel. (2) In pediatric thyroid carcinoma, chromosomal rearrangement (53%) was more common than point mutation (33%). (3) *ETV6*-*NTRK3* fusion was identified in 18% of samples, and was associated with aggressive histologic features such as non-encapsulation, solid/insular/trabecular patterns, extensive glandular involvement, and thick tumor fibrosis [14]. Ballester et al. analyzed FFPE and FNA samples from 25 pediatric PTCs (age range 10–19 years, median 14 years) using the 50-gene Ion AmpliSeq Cancer Hotspot Panel v2 on the Ion Torrent PGM sequencer [15]. No additional mutations were detected using the NGS assay on pediatric PTCs that initially were negative for *BRAF* V600E mutation, *RET/PTC1/3* fusion, and *TERT* promoter mutation [15].

### Follicular carcinoma

Follicular carcinoma (FC) is a well-differentiated thyroid carcinoma and the second-most common thyroid cancer after PTC. It accounts for 10% of the total thyroid cancers [16]. Although it is not firmly established, FC is classified into the minimally invasive type and the widely invasive type, according to the microscopic tumor extent [17]. It can be classified into the conventional type and the oncocytic type (Hurthle cell type) on the basis of cell type [18].

A previous study retrospectively collected FFPE or fresh frozen tissue samples from 36 FCs to study 12 cancer genes and 34 amplicons using the ThyroSeq panel on the Ion Torrent PGM sequencer. The analysis detected mutations of *NRAS* (*N* = 9), *KRAS* (*N* = 2), *HRAS* (*N* = 1), *TSHR* (*N* = 4), *TP53* (*N* = 4), and *PTEN* (*N* = 1) [10]. Interestingly, conventional type (*N* = 18) and oncocytic type (*N* = 18) samples showed distinguished genetic alterations. In the conventional type FCs, *NRAS* was the most frequently affected gene, followed by *TSHR* and *KRAS*. *TP53* was the most commonly mutated gene in the oncocytic type FCs, followed by *HRAS*, *KRAS*, and *PTEN* [19].

Swierniak et al. analyzed 26 follicular adenomas, 22 FCs, and 34 paired normal thyroid tissue samples. Targeted NGS of 372 genes using the TruSeq kit on the Illumina HiSeq 1500 platform yielded the following results [20]: (1) Somatic alterations were identified in oncogenes (*MDM2*, *FLI1*), transcription factors and repressors (*MITF*, *FLI1*, *ZNF331*), epigenetic enzymes (*KMT2A*, *NSD1*, *NCOA1*, *NCOA2*), and protein kinases (*JAK3*, *CHEK2*, *ALK*). (2) Single nucleotide variants were the most common types of mutations, and large structural variants were the least frequent. (3) A novel translocation in *DERL*/*COX6C* was detected. (4) Somatic alteration affected non-coding gene regions and exhibited high penetrance. These results suggest that FC has significant molecular heterogeneity, since FC reveals far more complex somatic alterations than PTC, and each tumor harbors distinct somatic alterations.

### Poorly differentiated carcinoma and anaplastic carcinoma

Poorly differentiated carcinoma (PDC) and anaplastic carcinoma (AC) are rare types of thyroid carcinoma, each with a prevalence of 10% [21, 22], and a 1–2% among all thyroid carcinomas [23]. PDC and AC have poor prognoses, with a 5-year survival rate of 51% and 0%, respectively [24]. Since these types of cancers respond poorly to conventional treatment options (including radioiodine therapy, chemotherapy, and radiotherapy), there are already clinical trials for molecular-targeted agents underway [25, 26]. Using NGS, it may be possible to identify targetable gene alterations that can improve the course of patient treatment.

In a study using the ThyroSeq panel on the Ion Torrent PGM sequencer, 12 genes with 34 amplicons were analyzed from FFPE or fresh frozen tissue from 10 PDCs and 27 ACs. The study showed that 30% of PDCs had mutations, whereas 74% of ACs had mutations [10]. The altered genes were *NRAS, PIK3CA, GNAS,* and *BRAF* in PDCs, and *TP53, BRAF, RAS, PIK3CA, PTEN,* and *CTNNB1* in ACs.

Sykorova et al. analyzed fresh frozen tissue samples from 3 PDCs and 5 ACs using the TruSight Cancer Panel, targeting 94 cancer-related genes on the Illumina MiSeq sequencer [27]. All PDCs and ACs showed more than one genetic alteration, and *TP53* mutations were identified in all but 2 cases [27]. *CDH1, FANCD2, CHECK2, ADH1B, GPC3, TP53,* and *PTEN* genes were altered in PDCs, and *ATM, HNF1A, MET, NF1, TP53, PTEN, MSH2, RB1, NBN, NF1, MUTYH, TSC2, HRAS,* and *EGFR* genes were altered in ACs [27]. However, the study could not assess mutation of larger genes or chromosomal rearrangements in a panel of 94 known cancer genes, nor could it distinguish germline mutations among the detected genetic changes.

Landa et al. performed NGS using the MSK-IMPACT cancer exome panel, and analyzed 341 genes from FFPE (*N* = 80) or fresh-frozen tissue (*N* = 37) samples from 34 PDCs and 33 ACs [28]. The analysis revealed the following: (1) ACs showed a higher mutation number than PDCs (6 ± 5 vs. 2 ± 3, median ± interquartile range), and harbored a higher frequency of mutation in *TP35, TERT* promoter, *PI3* *K/AKT/mTOR* pathway effector, *SWI/SNF* subunit, and histone methyltransferase. (2) In PDCs, clinicopathologic features were different based on the genetic alterations: 92% of *RAS* mutations were found in PDCs met the Turin criteria, whereas 81% of *BRAF* mutations were found in PDCs met the Memorial Sloan-Kettering Cancer Center (MSKCC) criteria. PDCs harboring *BRAF* mutations were smaller and had frequent nodal metastasis, whereas *RAS*-mutant PDCs showed larger tumor sizes and a higher rate of distant metastasis. (3) The association of mutation between *EIF1AX* and *RAS* was notable in both PDCs and ACs. *EIF1AX* mutations have been reported in approximately 1% of PTCs, and known to be mutually exclusive for *BRAF* and *RAS* mutations. However, *EIF1AX* mutations were found in 11% of PDCs and 9% of ACs, and 93% were associated with *RAS* mutations. (4) Chromosomal rearrangements (including *RET/PTC*, *ALK*, and *PAX8*-*PPARG* fusions) were found in 14% of PDCs, but were absent in ACs.

### Medullary carcinoma

Medullary carcinoma (MC) is a neuroendocrine tumor originating from C-cells, and accounts for approximately 5% of total thyroid cancers [29]. MC is composed of 75% sporadic form and 25% hereditary form, the latter resulting from a *RET* proto-oncogene mutation [30, 31].

Nikiforova et al. analyzed 12 genes and 34 amplicons using the ThyroSeq panel on the Ion Torrent PGM sequencer with FFPE or fresh frozen tissue samples from 15 sporadic MCs. Mutations were identified in 11 (73%) MCs, of which 7 (47%) were *RET* mutations, 3 (20%) were *HRAS* mutations, and 1 (7%) was a *KRAS* mutation [10]. Simbolo et al. analyzed 50 cancer-related genes using the Ion AmpliSeq Hot Spot Cancer Panel v2 on the Ion Torrent PGM sequencer. Out of 20 retrospectively collected FFPE samples of MCs, the study found that 85% of MCs harbored mutations as follows: 13 *RET* mutations (60%), 3 *HRAS* mutations (15%), 1 *KRAS* mutation (5%), 1 *STK11* mutation (5%), and 3 samples where mutations were undetected (15%) [32]. *RET* status was evaluated with both the NGS and Sanger sequencing methods, and NGS showed higher sensitivity than the Sanger method. NGS identified an additional 3 *RET* mutations, which were undetected by the Sanger method. Although multiple mutations were found in MCs by the NGS assay, a relevant therapeutic target has not been identified, and further investigation is required to improve the treatment of MCs.

## Limitation of NGS technology for thyroid cancer

One of the most profound limitations of applying NGS for thyroid cancer is a lack of sufficient evidence-based framework applicable to the clinical practice. As discussed in this review, the number of existing studies using NGS to analyze thyroid cancer stands at less than 15. Also, most of the previous studies were carried out at single institutes using specific subtypes of thyroid cancer in small sample sizes, rather than all types of thyroid cancers; therefore, resulting data would still be insufficient for making decisions on either patient diagnosis or treatment in clinical practice. To overcome such limitations, a large-scaled, global, and multicentered NGS study for thyroid cancer is required.

In a thyroid nodule with indeterminate cytology and *BRAF* V600E detected by NGS, surgical resection would be the most appropriate treatment option, since *BRAF* V600E is a highly specific mutation for PTC [13]. In contrast, *RAS* mutation can be detected in FVPTC and follicular neoplasm that require surgical excision, while also being present in benign adenomatous nodules [33] that do not require excision; therefore, further studies to identify the optimal treatment plan specific to mutation is needed. Apart from mutational variant, inadequate sample preparation, of both poor quality and quantity, can lead to false negative results. In samples with low tumor purity and small amount of DNA, low coverage would not be able to detect in allele with low frequency. Although DNA quality of cytology specimen is better than that of FFPE tissue, cytology specimens would contain some amount of normal tissue component. Also, evaluation of tumor purity may be an essential step before DNA preparation, particularly if the target nodule is small or an inexperienced person performs the aspiration procedure.

In addition to well-known *BRAF*, *RAS*, and *RET* mutations, NGS technology facilitated detection of new somatic alterations in thyroid cancer such as *MITF, MDM2, JAK3, FLI1, IDH1* etc. [20], all in which the significance of thyroid cancer has not been delineated yet. Larger scales of integrated genomic and phenotypic database should be provided to interpret NGS results. Also, an appropriate reporting system for NGS results in thyroid cancer is needed. Results from NGS analysis may encompass multiple variants, and each variant may have different clinical and biological significance. An appropriate tier system, with specific level of evidence, is required for reporting NGS results. Working group with large expertise to build a consensus guideline for reporting NGS results in thyroid cancer is requested. Besides intrinsic limitation of NGS platform, such as low detection rate of large indels, annotation errors of pipeline can be present. Clinicopathologic correlation and additional knowledge-based review of NGS report are essential for result interpretations. Currently, agents specifically targeting defined mutations are available, and patients who have the targetable mutation can benefit from optimized treatment and avoid unnecessary therapy. Inclusion of potential therapeutic target genes in the gene panel of targeted NGS, as well as the accumulation of information to build up database for future investigation, would also be required.

Most of NGS panels applied in thyroid cancer studies cover hotspot mutations, and are highly sensitive for evaluation of limited regions of selected genes; however, relevant mutation could be missing if not appropriately mapped. As described in previous studies, targeted NGS with specific gene panel showed high PPV. However, NGS analysis using 7-gene panel in previous study showed that 30–35% of thyroid cancer patients were still negative for mutation, and low NPV would require diagnostic surgery in benign nodules to prevent missing cancers [34]. High sensitivity of NGS technique showed that subclones within a nodule with mutations leading to aggressive clinical behaviors might be detected with low allele frequency [35]. Clinicians would face a dilemma with such cases, regarding whether to follow up with their patients or to refer them to surgical resection. Although different platforms and variant-calling pipelines were proven to have high concordance and sensitivity, detected mutations would be different in cases with tumor heterogeneity [36]. Genetic alteration of tumor results in tumor heterogeneity, which can be divided into intertumor heterogeneity that shows different genetic alterations based on the tumor sites, and intratumor heterogeneity that contains different genetic alterations within a same tumor. Concepts of aggressive clone and tumor heterogeneity are also present in thyroid cancer [37–39]. Furthermore, complicated situations may derive from NGS results, depending on whether NGS for each tissue sample was performed at relevant site and relevant time, and this can lead to repeated aspiration and/or biopsy. Also, in metastatic cancer, tissue samples should be obtained from metastatic sites; however, sites such as the brain or a specific bone are challenging for proper tissue sampling.

## Future of NGS for thyroid cancer

The fast-evolving NGS technology offers a cost-effective approach for cancer genomics, as well as in thyroid cancer. In future prospects of thyroid cancer, NGS can be used to detect circulating tumor cells or cell-free plasma DNA to identify early relapse and/or residual disease. Previous studies reported presence of circulating tumor cells or cell-free plasma DNA in thyroid cancer patients [40, 41], which appeared to be future candidates for NGS application. Furthermore, NGS can detect tumor-specific genetic alterations, which are used in follow-ups for patient monitoring. In patient monitoring, genetic alterations should be present in all tumor cells, while consistently and sustainably existing from both tumor development and during tumor progression. In thyroid cancer, *BRAF* V600E is the most common and the earliest genetic event in PTC [42, 43], and it appears to be a good candidate gene for monitoring. Also, during radioactive iodine and/or drug treatment of thyroid cancer, new mutation variants other than primary tumor can be recognized in NGS analysis of either circulating tumor cells or cell-free plasma DNA. This concept can be applied in identifying genetic alterations related to an acquired resistance to treatment during clinical course. Currently available studies of NGS application in thyroid cancer tend to focus on evaluating genetic alterations in specific types of thyroid cancer. In future research, an improved high-throughput pipeline should be used for a more comprehensive analysis of gene expression and DNA binding activity. In addition, a systems biology approach would also help discover the interaction and casual relationship between genes and/or proteins, introducing a new ground of thyroid cancer biology.

## Conclusions

The emergence of NGS technology has provided in-depth analysis of multiple, diverse cancers by a number of devices and gene panels, and has led to more effective options for cancer screening, prevention, diagnosis, prognosis, and targeted therapy. The use of NGS to study thyroid cancer has improved our understanding of the molecular genetics of thyroid cancer. In thyroid nodules of indeterminate cytology, such as FN/SFN and AUS/FLUS, the NGS test detected multiple genetic alterations and identified patients with a high risk of malignancy. Risk stratification using molecular signatures offers many more precise treatment options during patient management. The application of NGS for PTC, FC, MC, PDC, and AC revealed novel genetic alterations which were not detected by past sequencing methods. Newly discovered genetic alterations include genes associated with tumor recurrence and distant metastasis, which are candidates for molecular prognostic markers. However, limitations are also present with NGS, arising from variable sample types, multiple platforms and gene panels, and variable analysis programs, each of which can confound results. Standardization for quality control and the data-analytic process is needed to minimize the discrepancies between analyses. For poor-prognostic histologic types of thyroid cancer—MC, PDC, and AC—NGS studies identified several novel genetic alterations, but drug-actionable target genes have not been identified yet, and further investigation is required. Nevertheless, development of new sequencing technologies, such as NGS, enhances the cancer genome body of knowledge, and allows for more effective cancer screening, prevention, diagnosis, and monitoring. This in turn provides for better precision medicine and more curative cancer treatments.

## Abbreviations

NGS: next-generation sequencing
FNA: fine needle aspiration
PTC: papillary thyroid carcinoma
AUS/FLUS: atypia of undetermined significance/follicular lesion of undetermined significance
FN/SFN: follicular or oncocytic (Hurthle cell) neoplasm
SUSP: suspicious for malignant cells
PPV: positive predictive value
NPV: negative predictive value
TCGA: The Cancer Genome Atlas
FVPTC: follicular variant papillary thyroid carcinoma
FC: follicular carcinoma
PDC: poorly differentiated carcinoma
AC: anaplastic carcinoma
MSKCC: Memorial Sloan-Kettering Cancer Center
MC: medullary carcinoma
